# Highly aggressive rat prostate tumors rapidly precondition regional lymph nodes for subsequent metastatic growth

**DOI:** 10.1371/journal.pone.0187086

**Published:** 2017-10-26

**Authors:** Kerstin Strömvall, Marie Lundholm, Elin Thysell, Anders Bergh, Sofia Halin Bergström

**Affiliations:** Department of Medical Biosciences, Pathology, Umeå University, Umeå, Sweden; Southern Illinois University School of Medicine, UNITED STATES

## Abstract

The aim of this study was to examine in what ways MatLyLu (MLL) rat prostate tumors with high metastatic capacity influence regional lymph nodes prior to metastatic establishment compared to AT1 rat prostate tumors with low metastatic potential. MLL or AT1 tumor cells were injected into the ventral prostate of immunocompetent rats. Tumor and lymph node morphology, and lymph node mRNA expression of macrophage associated markers, T-cell associated markers, and cytokines were examined over time until the first microscopic signs of metastases (at day 14 for MLL- and at day 28 for AT1-tumors). Already at day 3 after tumor cell injection, when the tumors were extremely small and occupied less than 1% of the prostate volume, MLL- and AT1-tumors provoked different immune responses in both the prostate and the regional lymph nodes. MLL-tumors induced expression of immunosuppressive cytokines, suppressed T-cell accumulation, and directed T-cells towards an immunosuppressive phenotype. AT1-tumors caused a response more similar to that in vehicle-injected animals, with accumulation of T-cells in tumors and regional lymph nodes. Prostate tumors with high metastatic potential were able to precondition regional lymph nodes to subsequent metastatic growth in ways different from tumors with less metastatic potential. This may indicate the existence of a time-window when pre-metastatic changes in regional lymph nodes can aid in the prognostication of locally aggressive and potentially metastatic prostate cancer.

## Introduction

In order to grow and spread neoplastic cells need to interact successfully with adjacent and more remote cells, tissues and organs [1–7]. Consequently, this implies that locally aggressive cancers with high metastatic potential, already early and before the establishment of metastases, can influence adjacent cells and distant organs differently than tumors with low metastatic potential. Increased understanding of alterations induced in other organs before metastasis could therefore be used to improve both the early diagnosis and treatment of potentially metastatic prostate cancer [8–11].

Implantation of Dunning rat prostate cancer cells into the prostate of immunocompetent and syngeneic rats resulted in adaptive changes in major parts of the non-malignant prostate tissue [8, 10, 11]. The nature and magnitude of these changes were related to tumor aggressiveness and metastatic capacity [9, 11, 12]. Similar changes in the benign parts of the prostate were associated with disease aggressiveness and outcome in prostate cancer patients [9, 10, 13, 14]. Such adaptive changes are by us named TINT-changes, where TINT stands for tumor instructed (and thus indicating) normal (non-malignant) tissue.

Although pre-metastatic changes in lymph nodes (LNs) are well established for other tumor types [7, 15–17], it is largely unknown if regional LNs are reprogrammed/”tinted” also by the presence of potentially metastatic prostate cancers. By using two different types of orthotopic rat prostate tumors–the locally aggressive AT1-tumor with poor metastatic ability and the locally aggressive MatLyLu (MLL)-tumor with high metastatic ability [18]–we compared the global gene expression profiles in tumor tissue, in the surrounding benign parts of the prostate (TINT) and in regional LNs [11]. In animals with MLL-tumors, the gene expression profile, and morphological examination of the benign prostate tissue, suggested a tumor-promoting inflammatory response dominated by M2-macrophages [9, 11]. Furthermore, the gene expression profile in pre-metastatic regional LNs in animals with MLL-tumors was associated with decreased antigen presentation and immunosuppression [11]. In animals with similar sized AT1-tumors, the gene expression profiles in tumor tissue and regional LNs were instead associated with an activated anti-tumor immune response, and in the benign prostate tissue it was associated to inhibition of tumor growth and metastasis. [11].

These findings suggest that prostate cancer aggressiveness could potentially be evaluated by examining tumor-induced pre-metastatic changes in the regional LNs. The aim of this study was to compare temporal changes in regional LNs in animals with intra-prostatic AT1- or MLL-tumors and to explore at what time point and stage of development metastatic prostate tumors specifically influence regional LNs.

## Materials and methods

### Ethics statement

Immunocompetent and syngeneic adult Copenhagen rats (Charles River, Sulzfeld, Germany) were used in all experiments in this study and all animal work was carried out in accordance with protocols approved by the Umeå Ethical Committee for animal research (permit number A 42-15A). Animal maintenance, anesthesia, and euthanasia were performed as previously described [11] and strong efforts were made to minimize animal discomfort and suffering. In brief, anesthesia used before tumor cell injection and sacrifice was performed by intraperitoneal injections of Ketamine (75 mg/kg) and Medetomidine (0.5 mg/kg). At sacrifice, euthanasia was performed by anesthetizing the animals and then remove the heart. All animals included in the study survived to the endpoint of the experiment. The end-point was when tumors started to outgrow the prostate and morphological examinations showed presence of LN metastases. However, these tumors did not give rise to any symptoms.

### Experimental prostate cancer model

The Dunning R-3327 prostate tumor model consists of a series of transplantable rat prostate tumor cells lines that are all derived from a spontaneous tumor in the dorsolateral prostate of a Copenhagen rat [18]. From this series, AT1 and MLL rat prostate tumor cells were chosen for this study. Both these cell lines can be injected into the prostate of immunocompetent Copenhagen rats and form tumors that are fast growing and poorly differentiated. The main difference is that MLL-tumors have a higher ability to metastasize to regional LNs and lungs compared to AT1 [18].

AT1 and MLL cell lines were purchased from European Collection of Cell Cultures (ECACC, Sigma Aldrich; MLL # 94101454, AT1 # 94101449) and were grown in RPMI 1640 + GlutaMAX (Gibco) supplemented with 10% fetal bovine serum and 250 nM dexamethasone (Sigma Aldrich). 2 x 10^4^ AT1 cells, or 1 x 10^3^ MLL cells (suspended in 10 μl RPMI) were carefully injected into the right ventral prostate lobe as previously described [11]. Rats were sacrificed at 3 (AT1, n = 8; MLL, n = 8), 7 (AT1, n = 8; MLL, n = 8), 10 (AT1, n = 8; MLL, n = 9), 14 (AT1, n = 8; MLL, n = 7) and 28 days (AT1, n = 8) after tumor cell injection. The ventral prostate and the right and left iliac LNs were removed (at least 2 LNs/animal), weighed, frozen in liquid nitrogen, and stored in -80°C. These LNs were chosen, as they were the first to be bilaterally labeled when ink was injected into the right ventral prostate lobe (S1 Fig). Tissues from sham-operated and vehicle-injected rats (10μl RPMI) (n = 7-9/time point), or treatment-naïve rats (n = 8) were used as controls.

### Lymph node and prostate tumor morphology

Cryosections of tumor-, prostate- and LN-tissue were stained with primary antibodies against CD3 (#180102, Invitrogen, diluted 1:50), CD68 (Clone ED1, #MCA341R Serotec, diluted 1:200), and Ki67 (Clone SP6, #ab16667, Abcam, diluted 1:100) to visualize T-lymphocytes, macrophages and proliferating cells. “Envision HRP Rabbit” (#K4003, Dako) was used as secondary antibody for CD3 and Ki67, and “Mach3 Mouse on rat” (#MRT621, Biocare) was used as secondary antibody for CD68. The slides were developed using diaminobenzidine (Dako). The volume density of CD3^+^ and CD68^+^ was determined using stereological techniques as described earlier [9, 19]. In brief, this was done using a square-lattice mounted in the eye-piece of a light-microscope and counting the number of cross-sections falling on the immunostained cell type and on the unstained reference tissue space. Hematoxylin-and eosin-stained tissue sections were also examined and the volume density of polymorphonuclear leukocytes (PMNs, recognized by nuclear morphology) and the maximal tumor area was measured using the square-lattice described above. Values are expressed as mean +/- standard deviation and the Student’s T-test was used to compare groups using Statistic 12 (Statsoft Inc, Tulsa, OK).

### RNA extraction and qRT- PCR

Total RNA from LN tissue (10 μm sections, 30–60 sections/LN) was extracted using Allprep DNA/RNA/Protein mini kit (#80004, Qiagen) and quantified with Nanodrop-1000 spectrophotometer (version 3.8.1, Thermo Fisher Scientifics). 1.5 μg of total RNA was reverse transcribed into cDNA using SuperScript® VILO™ MasterMix (#11755, Invitrogen). Predesigned KiCqStart® SYBR® Green primers (S1 Table) were used and all primer-pairs were evaluated with standard curves to ensure that the amount of sample analyzed was within the dynamic range of the assays. qPCR was run on ABI 7900 HT instrument (Applied Biosystems) using the AQ standard cycling protocol plus melt curve analysis. Each qPCR reaction was run in duplicates and samples with duplicate Ct-values differing > 0.5 cycles were excluded. TATAA Interplate Calibrator for SYBR (IPC) (#IPC250S, TATAA Biocenter) was run in 4 replicates/plate (2x10^6^ copies of IPC-template/reaction). Melt curves and amplification plots were manually checked with the SDS 2.4.1 software (Applied Biosystems) to evaluate product specificity and quality of the Ct-measurements. Genex software (version 6.0.5.225, MultiD Analyses AB) was used for interplate calibration (using the mean IPC value for each plate), calculation of relative expression using the comparative Ct method, and statistical analysis. Average Ct-value for each sample was normalized using the reference genes *Rab14*, *Pi4kb*, and *RGD1311578*. mRNA expression in LN-tissue from left and right side was measured separately, but since no obvious difference could be detected, the average Ct-value of the right and left LN was used when calculating relative expression. Values are presented as log2-transformed mean expression +/- SEM relative to the treatment-naïve group. The Mann Whitney U test and the Student’s T-test was used when comparing groups, and a p-value < 0.05 in both tests was considered significant. The unsupervised multivariate projection method, Principal Component analysis (PCA), was also used to compare subgroups with respect to gene expression (SIMCA 14.0, Umetrics, Umeå, Sweden).

## Results

### Morphological analysis of orthotopic rat prostate tumors

Using an orthotopic prostate cancer model, we examined metastasis-free regional LNs (i.e. no tumor cells were detected by light microscopy) at different time points after tumor establishment in the prostate by analyzing morphology and mRNA expression of selected genes. Fast-growing MLL tumor cells with high metastatic potential, fast-growing AT1 tumor cells with low metastatic potential, or vehicle was injected into the right ventral prostate of immunocompetent Copenhagen rats and tumors and draining LNs were studied at day 3, 7, 10, 14, and for AT1 also at day 28, post injection.

**Fig 1:**
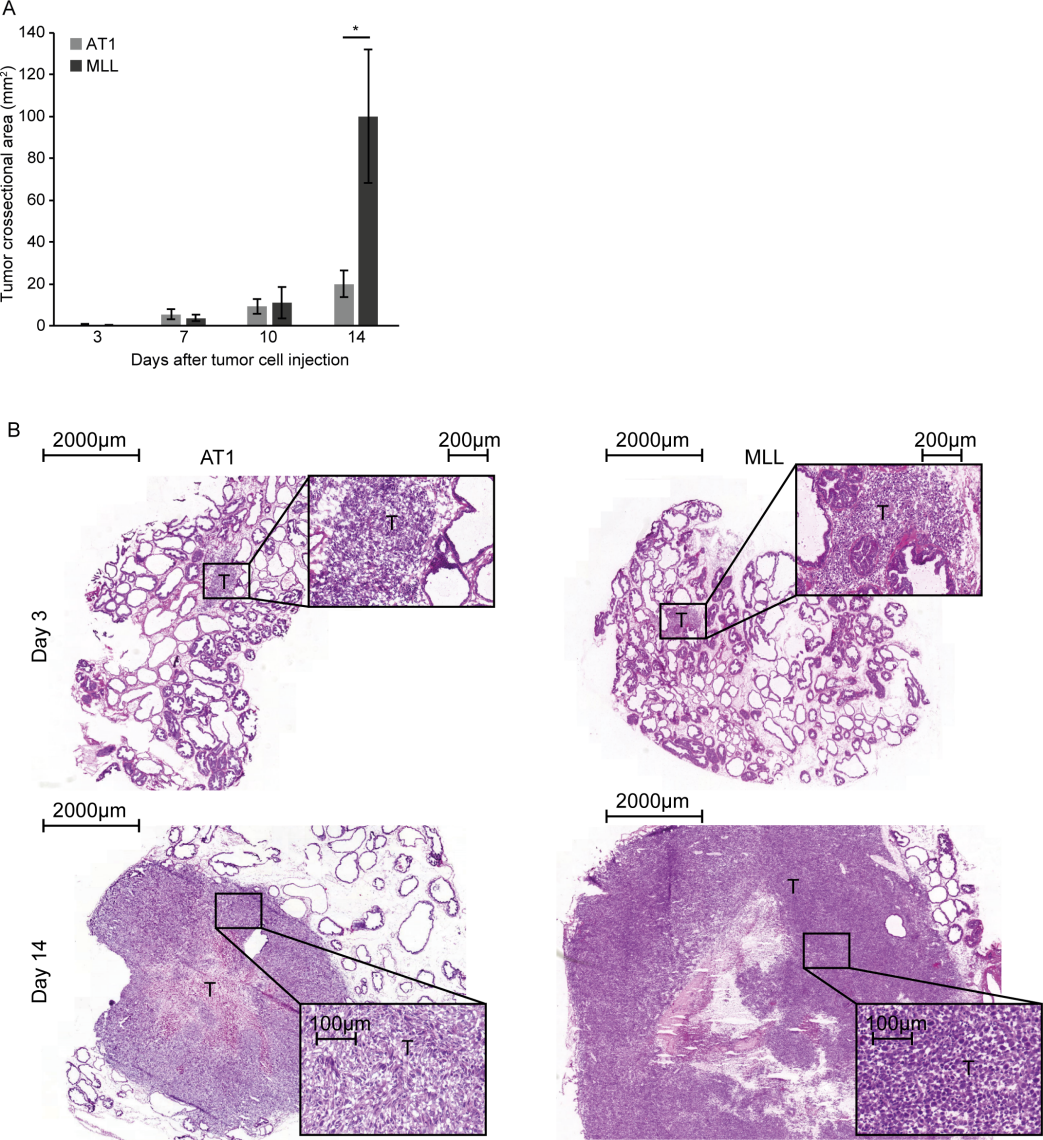
Tumor size. A) Tumor cross-sectional area at different time points post injection of 2x10^4^ AT1-, or 1x10^3^ MLL tumor cells into the ventral prostate (bars represent mean +/- SD, n = 7–8 animals/group, * p < 0.05). B) Representative eosin-hematoxylin stained sections showing intraprostatic AT1- and MLL-tumors at day 3 and 14 after tumor cell injection (T; tumor).

Tumor growth of both tumor types was rapid and the mean tumor cross-sectional area was not statistically different between AT1- and MLL-tumors until at day 14 after tumor cell injection (Fig 1A). Vehicle injection did not affect the weight of the right ventral prostate lobe at any time point studied compared to that in treatment-naïve animals (mean weight +/- SD in naïve was 0.16 +/- 0.03 g vs. vehicle day 3: 0.14 +/- 0.05, p = 0.42; day 7: 0.16 +/- 0.04, p = 0.85; day 10: 0.17 +/- 0.03, p = 0.35; and day 14: 0.17 +/-0.04, p = 0.41). At day 3, the tumors were small and occupied less than 1% of the prostate lobe volume (Fig 1B). At day 7 and 10 both tumor types were intermediate in size (occupying approximately 10% and 30% of the prostate lobe volume, respectively) and were still surrounded by normal prostate tissue. At day 14, MLL-tumors had markedly outgrown the injected prostate lobe, while AT1-tumors occupied approximately 50% of the prostate lobe volume (Fig 1B). At day 28, AT1-tumors had outgrown the injected prostate lobe, and the mean weight was similar to the mean weight of MLL-tumors at day 14 (1.3 +/- 0.4 and 1.0 +/- 0.4 g, respectively, p = 0.18, n = 7–8).

**Fig 2:**
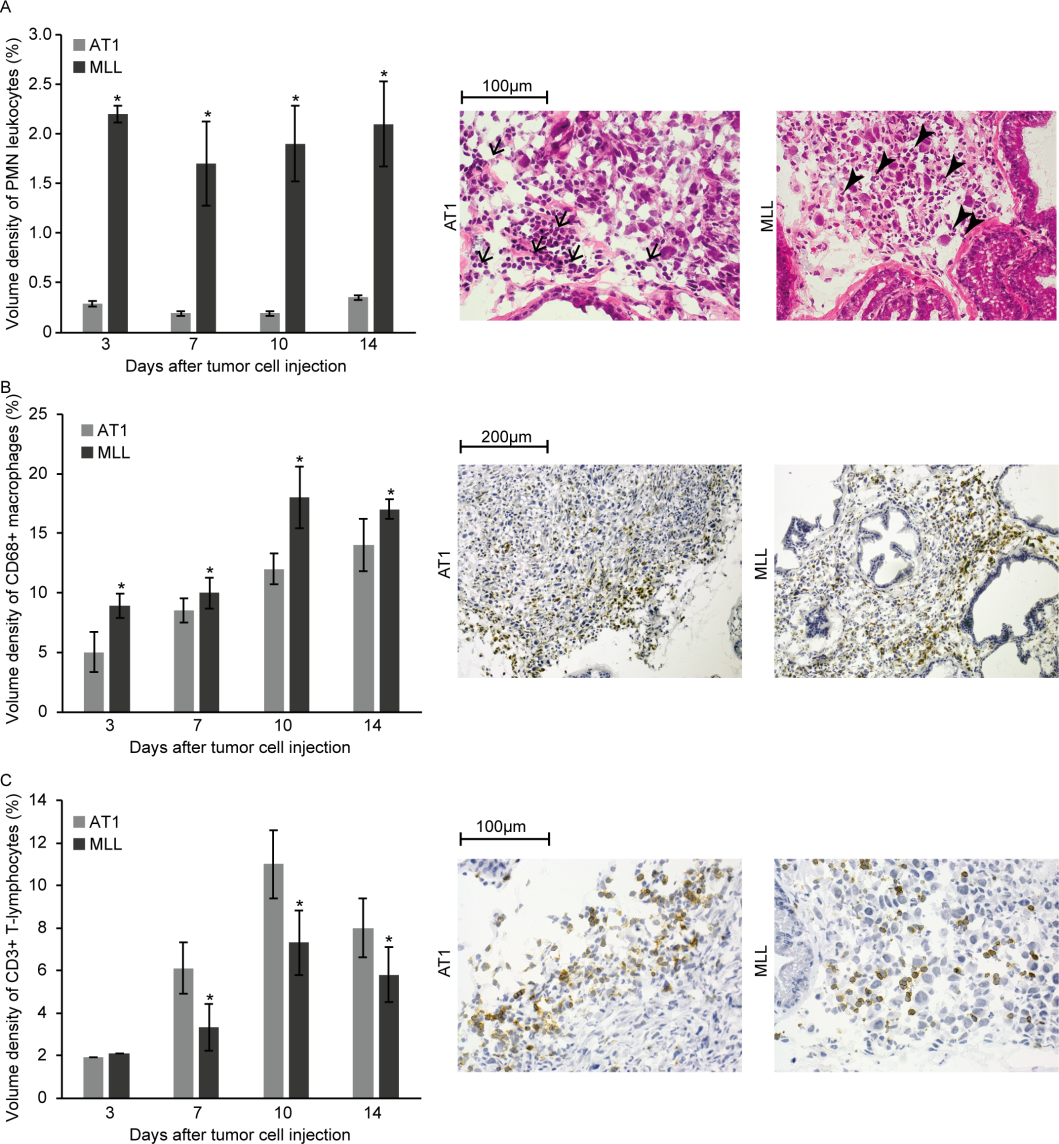
Inflammatory cells at the invasive zone of AT1- and MLL-tumors at different time points post injection of tumor cells. A) Volume density of polymorphonuclear leukocytes (PMNs) at the invasive zone. Representative eosin-hematoxylin stained sections showing AT1- and MLL-tumors at day 3 post tumor cell injection. Note the difference in the recruited inflammatory cells with mainly PMNs (arrowheads) in MLL-tumors, and lymphocytes (arrows) in AT1-tumors. B) Volume density CD68^+^ macrophages at the invasive zone. Representative sections showing CD68 staining (brown) in AT1- and MLL-tumors at day 3. C) Volume density of CD3^+^ T-lymphocytes in the tumor invasive zone. Representative sections showing CD3 staining in AT1- and MLL-tumors at day 7. Bars represent mean +/- SD, n = 7–8 animals/group, * p < 0.05.

At day 3, the fraction of neoplastic cells within the tumor masses was significantly lower in MLL-tumors (40 +/- 1.6%, n = 5) compared to AT1-tumors (67 +/- 7.5%, n = 5, p<0.05) with mostly infiltrating inflammatory cells constituting the remaining part. Intermingled between the neoplastic cells in MLL-tumors, we observed several CD68^+^ macrophages, PMNs and some lymphocytes (Fig 2). AT1-tumors generally grew as a small tumor mass with the tumor cells lying close to each other surrounded and invaded by inflammatory cells, mainly CD68^+^ macrophages, lymphocytes and some PMNs (Fig 2).

Metastasis to regional LNs from MLL-tumors, as from tumors in general, presumably occurs through lymph vessels at the tumor border [11, 20, 21]. Signals affecting the LNs are therefore likely to be derived from cells at the tumor border. We therefore examined the tumor invasive zone in more detail (defined as within 0.25 mm from the tumor border).

At all time points examined, the density of PMN-leukocytes at the tumor invasive zone was considerably higher in MLL- than in AT1-tumors (6 to 10-fold) (Fig 2A). Similarly, the density of CD68^+^ macrophages was higher in MLL- than in AT1-tumors (Fig 2B), but the difference was not of the same magnitude as for PMNs. From day 7, the density of CD3^+^ T-lymphocytes was higher in the invasive zone of AT1- than MLL-tumors (Fig 2C).

Taken together this suggests that tumor cells with different metastatic potential interact differently with the host and that this difference is established already when the tumors are very small.

### Morphological analysis of regional LNs

Substances injected into one ventral prostate lobe are known to spread bilaterally and to regional lymph nodes on both sides [22]. By injecting ink into the right ventral prostate we also found that iliac LNs on both the injected right side and the contralateral left side drained the ventral prostate (S1 Fig). LNs from the right (tumor-bearing) side were used for morphological quantifications while mRNA expression (see below) was examined in LNs from both the right and the left side.

**Fig 3:**
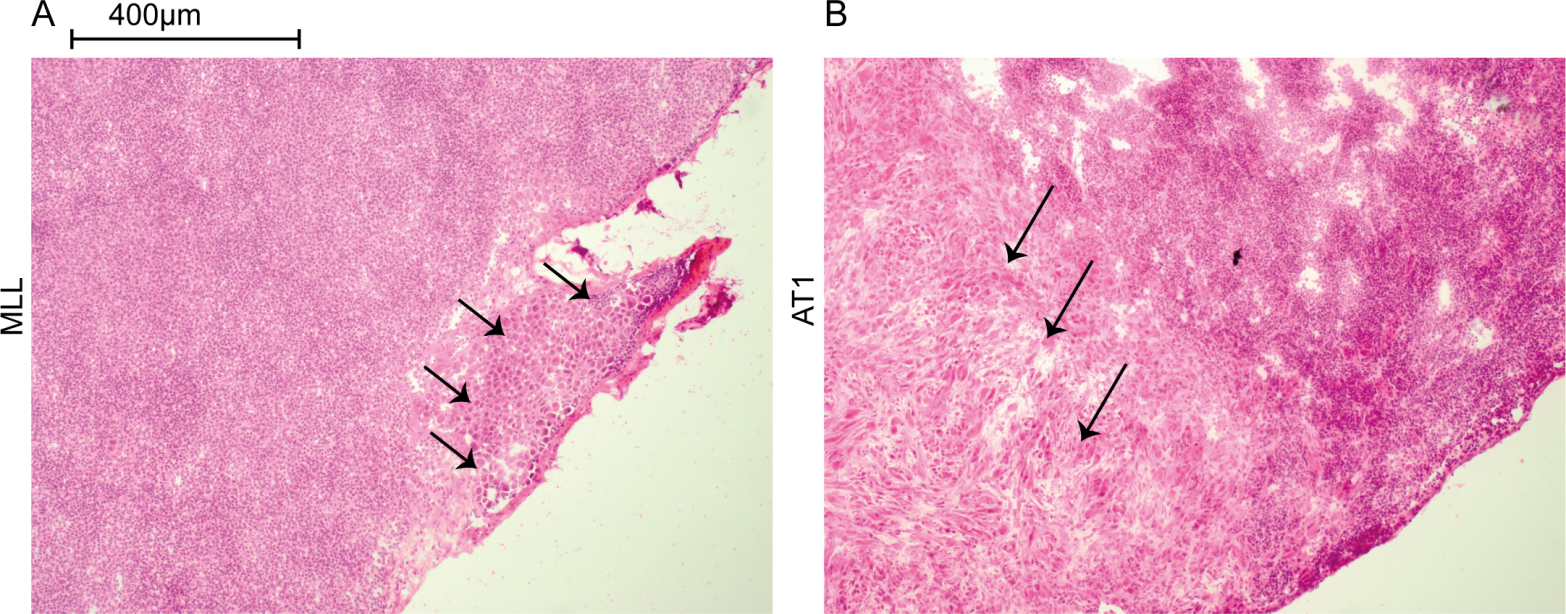
Lymph node metastases. Sections from regional lymph nodes from rats carrying a 14 day MLL-tumor (A) or a 28 day AT1-tumor (B). The first signs of micrometastases were detected at day 14 in the MLL-model (3 out of 7 animals) and at day 28 in the AT1-model (1 out of 8). Metastatic cells, first noted in the peripheral subcapsular parts of the nodes (arrows), were larger than other cells in the lymph nodes and therefore easily detected by light microscopy.

In animals with MLL-tumors, the first LN metastases were detected at day 14 when at least 3 out of 7 animals had microscopic lymph node metastases (Fig 3). In animals with AT1-tumors, LN metastases were not observed until day 28 when 1 out of 8 animals showed microscopic metastases (Fig 3).

**Fig 4:**
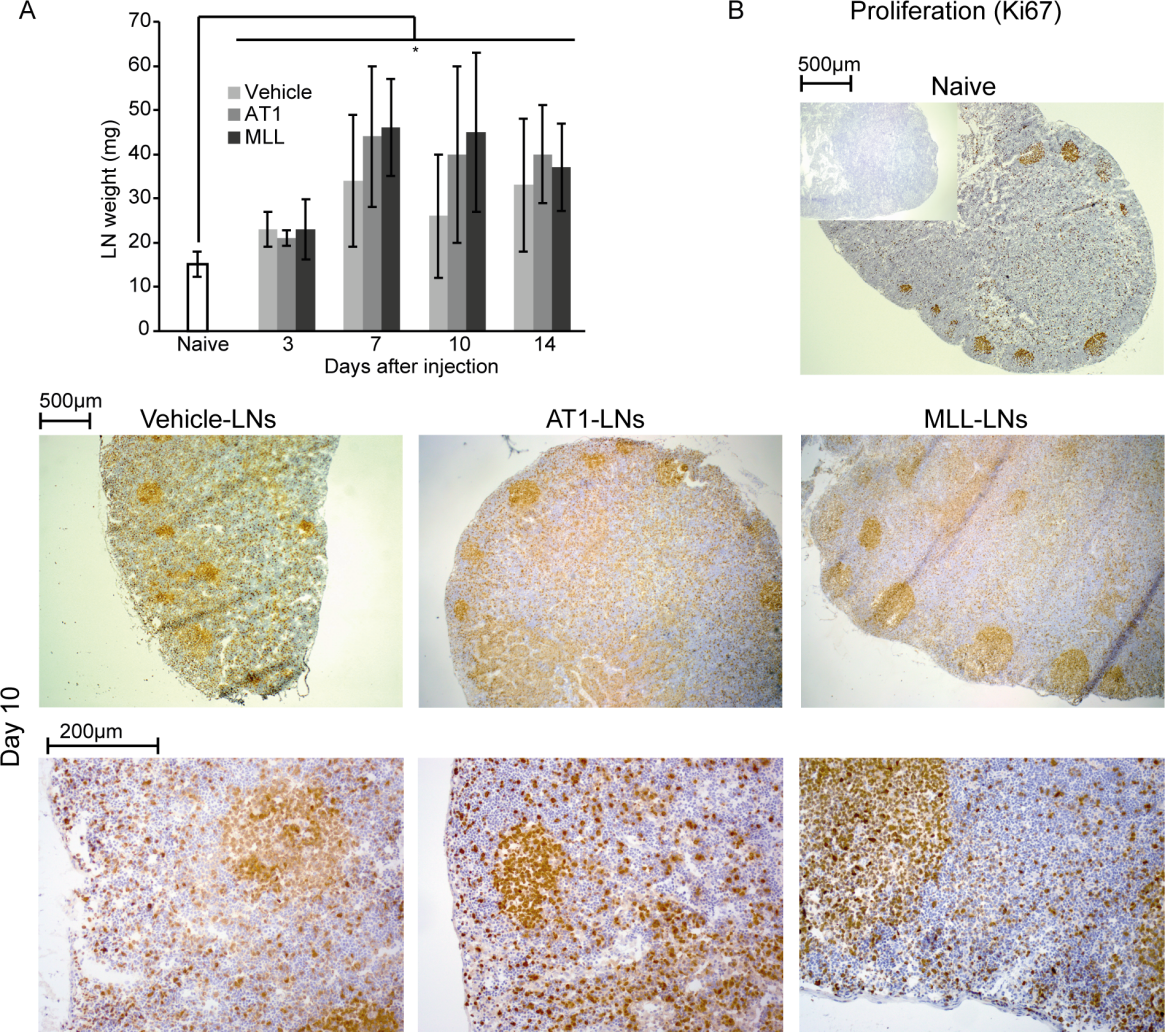
Lymph node weight and proliferation. A) Lymph node (LN) weight at different time points after injection of vehicle, AT1-, or MLL tumor cells into the prostate compared to LNs from treatment-naïve animals (bars represent mean +/- SD, n = 7–8 animals/group, * p < 0.05). B) Representative sections in two different magnifications showing proliferation (Ki67, brown) in vehicle-, AT1-, and MLL-LNs at day 10 compared to treatment-naïve LNs (insert shows control staining without primary antibody).

Abdominal surgery and vehicle injection into the prostate lobe increased the mean weight of the draining LNs from day 3 to day 14 compared to that in treatment-naïve animals (Fig 4A). A similar increase in LN weight was seen from day 3 and onwards in rats injected with AT1- and MLL tumor cells (Fig 4A). As the increase in LN weight was similar in all groups (Fig 4A), it could mainly be due to a systemic effect of the surgery required for injection. To discover tumor-induced morphological changes in the LNs we therefore compared LNs from tumor cell- injected rats to LNs from both vehicle-injected and treatment-naïve rats. Furthermore, to examine changes due to metastatic capacity we compared LNs draining MLL-tumors to LNs draining AT1-tumors.

All treatments caused an increase in LN weight suggesting a response associated with increased cell proliferation, which was also seen as increased Ki67 labeling in LNs from all treatment groups (Fig 4B). In animals with AT1-tumors, many of the proliferating cells were seen in para-follicular T-lymphocyte dominated areas (Fig 4B). In animals with MLL-tumors or vehicle-injected prostates, proliferating cells were seen both within lymph follicles, in parafollicular regions and in the medulla (Fig 4B) but this potential difference between tumor- types was not further explored.

**Fig 5:**
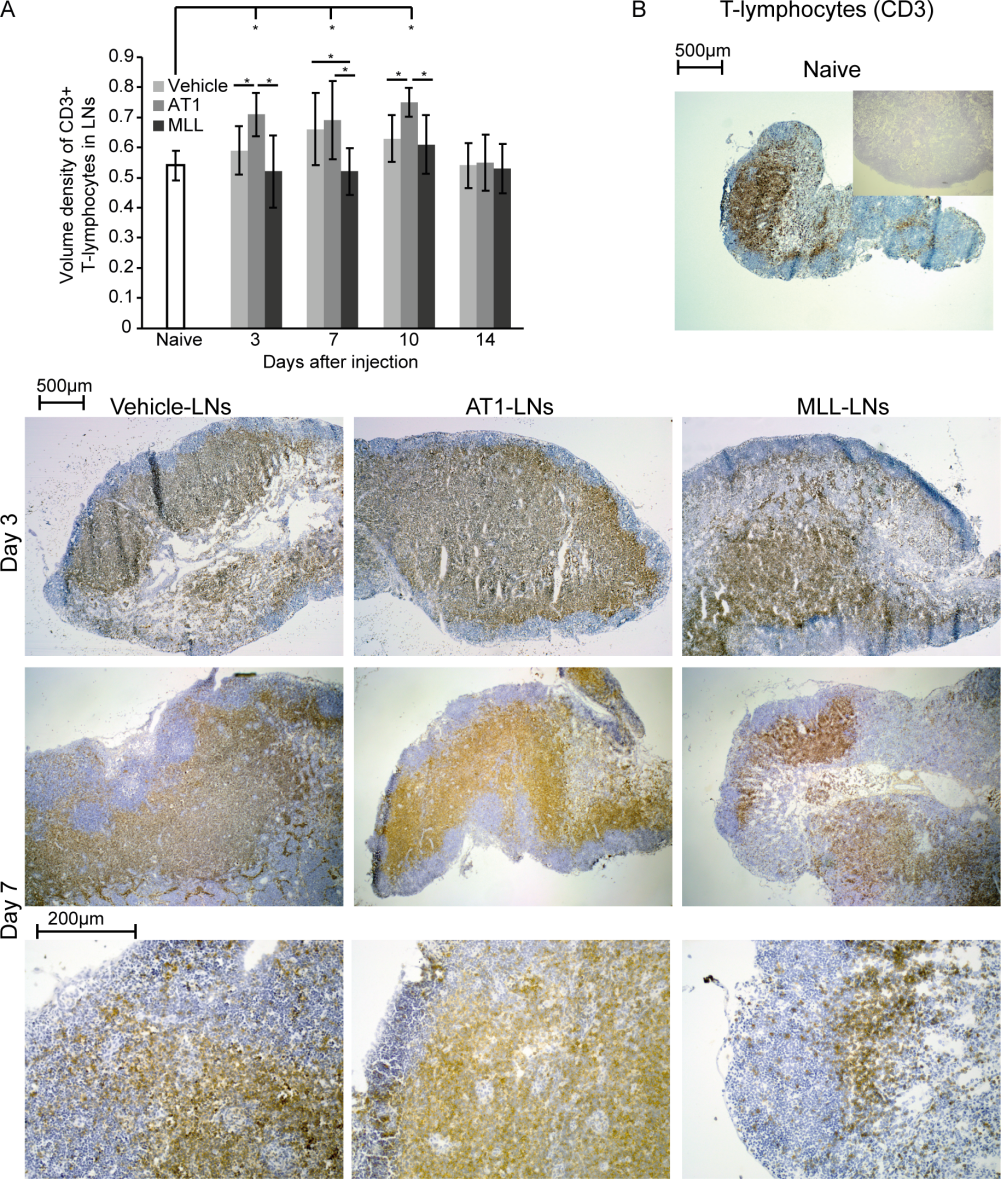
CD3^+^ T-lymphocytes in tumor-draining lymph nodes. A) Volume density of CD3^+^ T-lymphocytes in regional lymph nodes (LNs) at different time points post injection of vehicle, AT1-, or MLL tumor cells into the ventral prostate (bars represent mean +/- SD, n = 7–8 animals/group, * p < 0.05). B) Representative sections showing CD3 staining (brown) of vehicle-, AT1- and MLL-LNs at day 3 and 7 (two different magnifications) compared to treatment-naïve LNs (insert shows control staining without primary antibody).

The volume density of CD3^+^ T-lymphocytes in the regional LNs was slightly but not significantly increased by vehicle-injection into the ventral prostate (Fig 5). Like in the tumor invasive zone, AT1-LNs had a significantly increased density of CD3^+^ T-lymphocytes at day 3, 7, and 10 compared to treatment naïve LN-controls (Fig 5). In contrast, the density of CD3^+^ cells was unaffected in MLL-LNs compared to that in treatment naïve control-LNs (Fig 5). Subsequently, the density of CD3^+^ T-lymphocytes was also significantly higher in AT1-LNs compared to MLL-LNs at day 3, 7, and 10 (Fig 5).

### Temporal changes in mRNA expression in pre-metastatic regional lymph nodes

**Fig 6:**
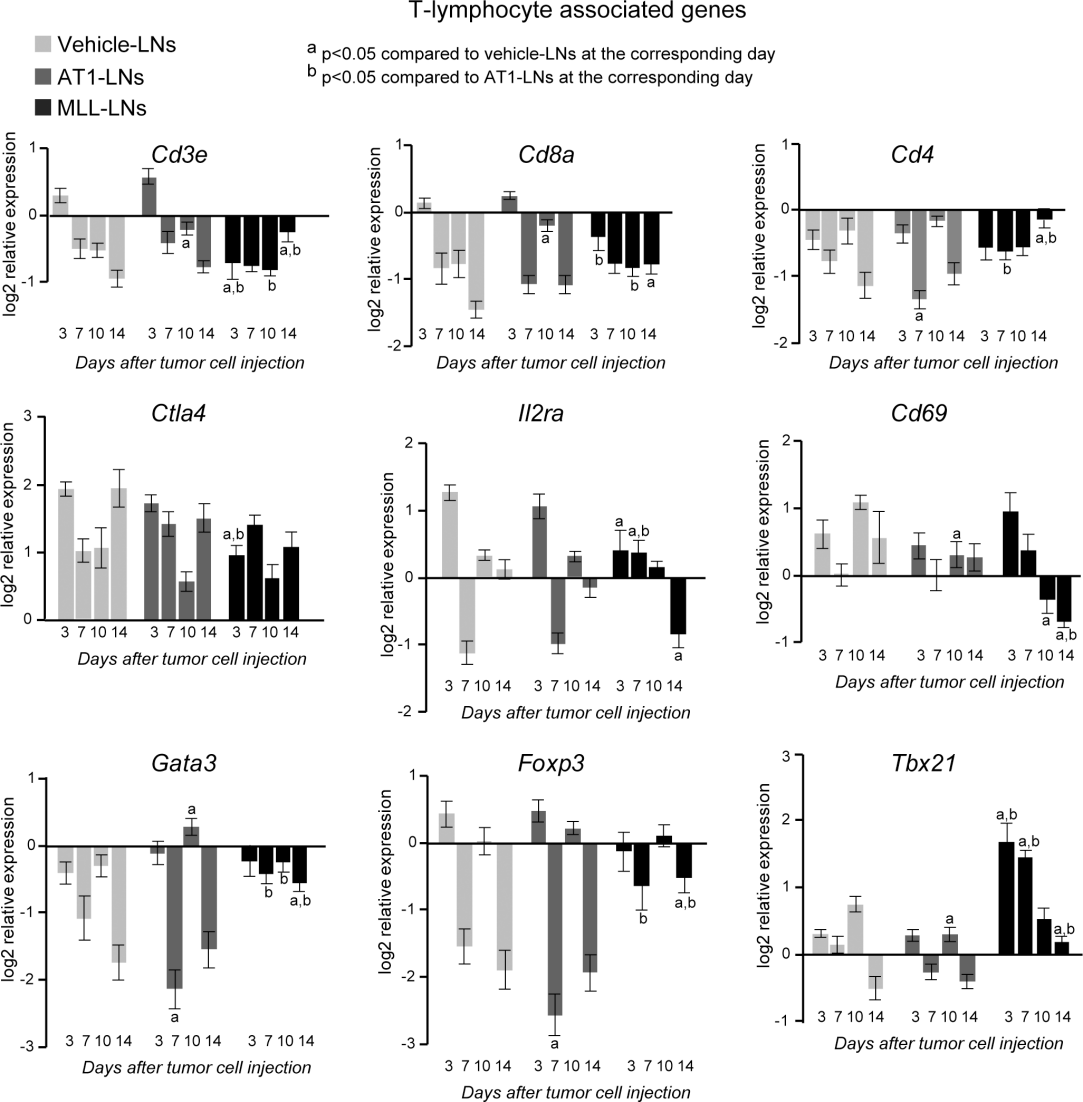
mRNA expression of T-lymphocyte markers in tumor-draining LNs. mRNA expression of T-lymphocyte associated markers in vehicle-, AT1-, or MLL-LNs relative to LNs from treatment-naïve animals (baseline) at day 3, 7, 10 and 14 after injection of tumor cells/vehicle into the prostate (bars represent log2 mean relative expression +/- SEM, n = 7–9 animals/group).

**Fig 7:**
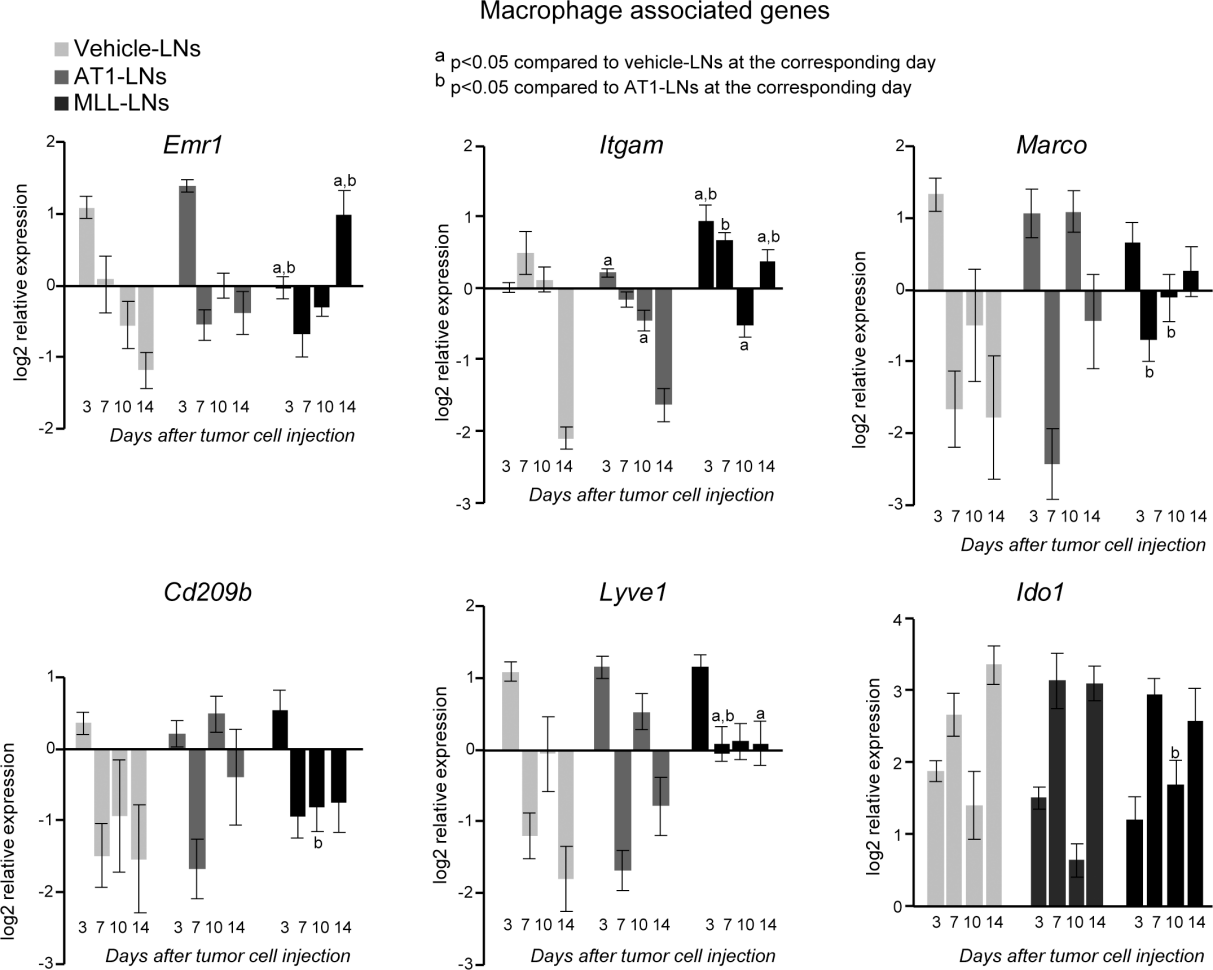
mRNA expression of macrophage markers in tumor-draining LNs. mRNA expression of macrophage associated markers in vehicle-, AT1-, or MLL-LNs relative to LNs from treatment-naïve animals (baseline) at day 3, 7, 10 and 14 after injection of tumor cells/vehicle into the prostate (bars represent log2 mean relative expression +/- SEM, n = 7–9 animals/group).

**Fig 8:**
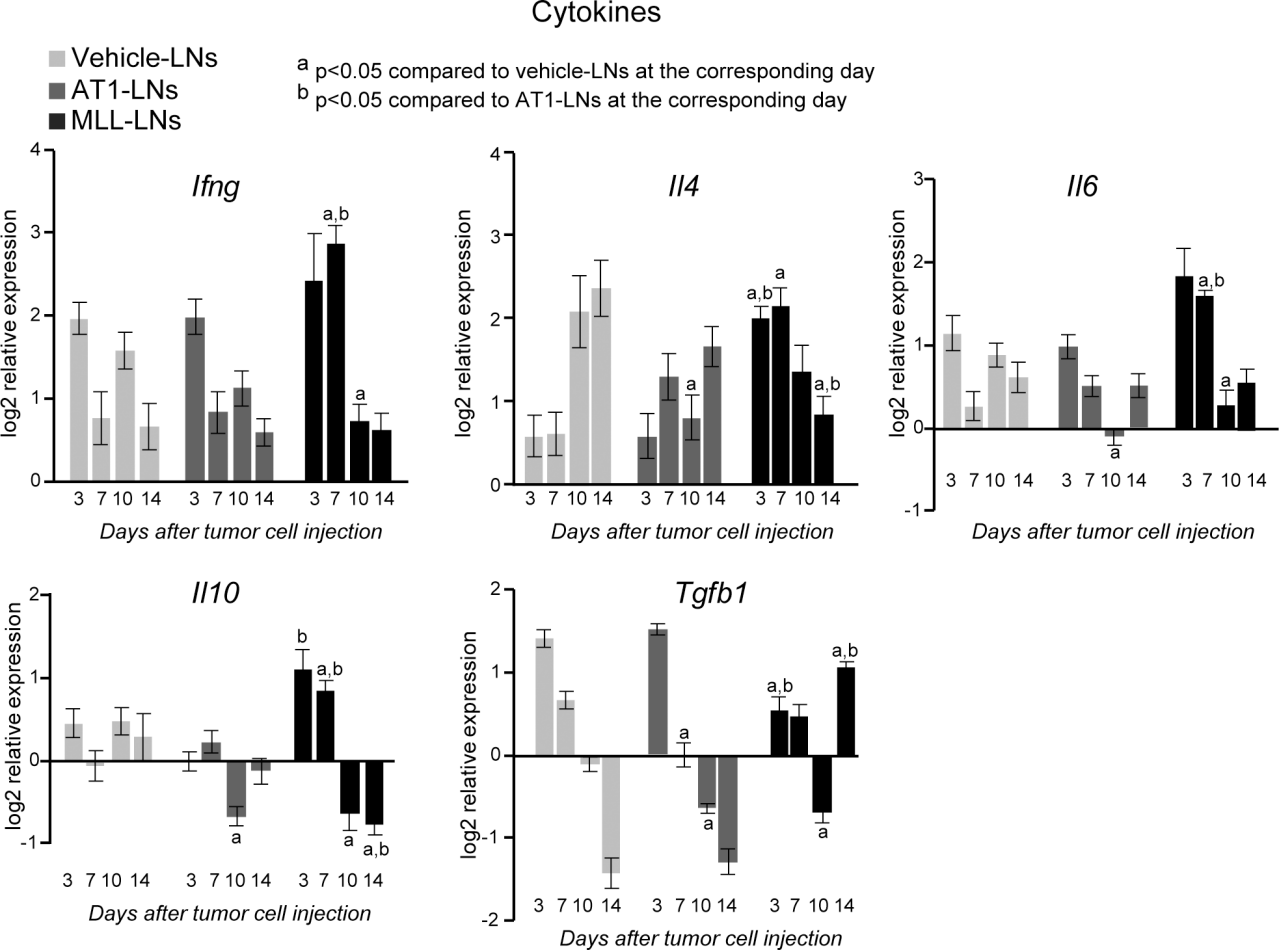
mRNA expression of cytokines in tumor-draining LNs. mRNA expression of cytokines in vehicle-, AT1-, or MLL-LNs relative to LNs from treatment-naïve animals (baseline) at day 3, 7, 10 and 14 after injection of tumor cells/vehicle into the prostate (bars represent log2 mean relative expression +/- SEM, naïve: n = 4–7, vehicle-, AT1, and MLL: n = 7–9 animals/group).

To explore tumor-induced immune responses in pre-metastatic LNs further we selected a panel of genes representing: 1) T-lymphocyte associated markers (*Cd3e*, *Cd4*, *Cd8a*, *Il2ra*, *Tbx21*, *Gata3*, *Foxp3*, *Cd69* and *Ctla4*), 2) macrophage associated markers (*Cd209b*, *Marco*, *Itgam*, *Emr1*, *Ido1 and Lyve1*), and 3) cytokines/growth factors (*Il10*, *Il6*, *Il4*, *Ifng*, and *Tgfb1*). As LNs on both sides drain the ventral prostate, each animal was represented by the mean expression of the right and left LN. Injection of vehicle into the prostate resulted in altered mRNA expression in regional LNs for most of the studied genes compared to treatment naïve animals (baseline in Figs 6–8, S2 Table). Vehicle-LNs therefore represent effects induced in the LNs due to injection and surgery.

#### Gene expression of T-lymphocyte associated markers

In general, the expression pattern over time for T-cell associated markers was comparable in AT1- and vehicle-LNs suggesting that the changes in gene expression in the AT1-LNs were similar to that induced by surgery (Fig 6). Conversely, the MLL-LNs had an expression pattern for these markers that was more distinct from the other groups (Fig 6).

The increased expression of *Cd3e* (T-cell receptor marker) and *Cd8a* (cytotoxic T-cell marker) at day 3 in AT1- and vehicle-LNs was not noticed in the MLL-LNs, but was instead decreased and had similar expression over time (Fig 6). This is in line with the CD3 staining showing decreased number of T-cells in the MLL-LNs compared to vehicle- and AT1-LNs (Fig 5). *Ctla4* (*Cd152*, co-inhibitor of T-cell activity) and *Il2ra* (*Cd25*, involved in T-cell and B-cell activation and expressed on regulatory T cells) were also higher in AT1- and vehicle-LNs compared to MLL-LNs at day 3, further suggesting a higher T-cell activity in AT1- and vehicle-LNs compared to MLL-LNs early after tumor cell/vehicle injection. However, the expression of *Cd4* (T-helper cell marker) was similar in all groups at day 3 while the expression of *Tbx21* (a transcription factor in T helper 1 cells) was significantly increased in MLL-LNs compared to vehicle- and AT1-LNs (Fig 6). At day 7, the expressions of *Il2ra*, *Cd4* and *Foxp3* (regulatory T-cell marker) were higher in MLL-LNs compared to AT1-LNs, suggesting a higher fraction of immunosuppressive regulatory T-cells.

14 days post injection was the first time point when microscopic metastases were detected in MLL-LNs (Fig 3), and therefore could the observed changes possibly be due to the presence of metastatic cells. At this time point the expression of *Cd3e*, *Cd8a*, *Cd4*, *Tbx21*, *Gata3* (T-helper 2 marker), and *Foxp3* were higher, while the expression of *Cd69* (early activation marker) and *Il2ra* was lower, in MLL-LNs compared to AT1- and vehicle-LNs (Fig 6).

#### Gene expression of macrophage associated markers

The expression pattern for the macrophage associated genes, *Itgam* (*Cd11b*), *Emr1* (*F4/80*) and *Lyve1* (also expressed on lymphatic endothelial cells) was similar over time for AT1- and vehicle-LNs, while the expression pattern in MLL-LNs was different compared to the other groups (Fig 7). For example, at day 3, *Itgam* expression was increased and *Emr1* (*F4/80*) expression was lower in MLL-LNs compared to AT1- and vehicle-LNs (Fig 7). At day 14, expression of *Itgam*, *Emr1*, and *Lyve1* were higher in MLL-LNs compared to AT1- and vehicle-LNs (Fig 7).

For the other macrophage markers examined, *Marco*, *Cd209b* and *Ido*, only minor changes in gene expressions were found between the groups, except at day 10 (Fig 7).

#### Gene expression of cytokines

Most of the significant changes were found when comparing cytokine expression in MLL-LNs compared to vehicle- and AT1-LNs (Fig 8), suggesting that cytokine expression is affected differently in animals having tumors with high metastatic ability. *Ifng*, *Il4*, *Il6*, and *Il10* were all higher in MLL-LNs compared to vehicle- and/or AT1-LNs at the earlier time points studied (day 3 and 7), while the expression of *Tgfb1* was lower (Fig 8). This suggests a broad cytokine increase in MLL-LNs early after tumor cell injection. However, at the later time points (day 10 and 14) the expression pattern was shifted with lower expression of *Ifng*, *Il4*, *Il6*, and *Il10* and higher expression of *Tgfb1* in MLL-LNs compared to the other groups (Fig 8).

#### Summary of mRNA expression patterns

Taken together, the gene expression pattern over time was often comparable in AT1- and vehicle-LNs suggesting that the response in AT1-LNs was similar to that induced by surgery. Notably, the MLL-LNs had an expression pattern that differed from the other groups, suggesting that tumors with high metastatic capacity affect draining LNs differently than tumors with low metastatic potential.

**Fig 9:**
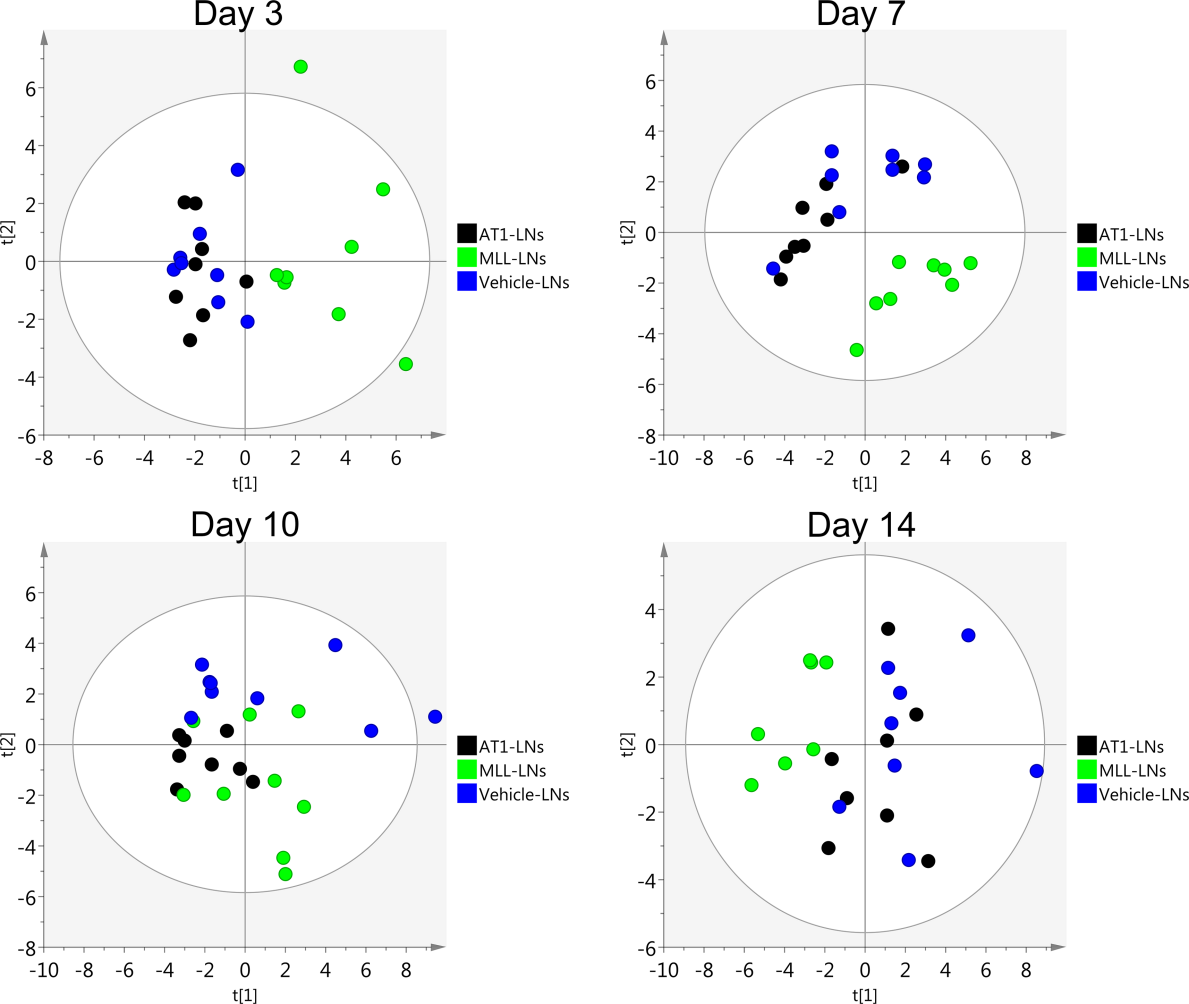
Principal component analysis. Score plots showing the variability explained by the 1^st^ and 2^nd^ components of a PCA-model constructed using log2 transformed relative gene expression values in vehicle-, AT1- and MLL-LNs at day 3, 7, 10 and 14 after injection of tumor cells/vehicle into the prostate (n = 7–9 animal/group).

Differences between MLL-LNs and AT1-LNs were detected already at day 3, when the tumors were small, but also at day 14 when microscopic metastases were found in the MLL-LNs (Fig 3). In addition, principal component analysis (PCA) of the expression data at day 3, 7, 10, and 14 showed that AT1- and vehicle-LNs clustered together while the MLL-LNs were more or less separated from the other groups (Fig 9).

## Discussion

If aggressive and potentially metastatic prostate cancer induces specific changes in regional LNs prior to metastasis this information could be of considerable diagnostic and therapeutic importance. Particularly if such changes are induced already when the tumors are small and curable and consequently that the time-window between the induction of pre-metastatic changes and metastatic colonization is sufficiently long. The current study suggests, at least in an animal model, that such potentially useful pre-metastatic changes could actually be present since 1) metastatic tumors induced specific pre-metastatic changes in regional lymph nodes and 2) some of these changes could be detected already when the tumors were surprisingly small.

Our previous study, examining global gene expression profiles at day 10 after tumor cell injection, suggests that MLL-LNs when compared to AT1-LNs is characterized by decreased quantity, differentiation, and activation of immune cells, and by signs of immune evasion [11]. For example, we found higher expression of *Ctla4* and *Lag3* in MLL-LNs that could inhibit anti-tumor immune responses [23–25]. Moreover, we detected lower levels of *Csf1/Csfr1*, *Clec1b*, *Osm*, *Tnfsf11* in MLL-LNs that could result in decreased numbers of antigen presenting cells and impaired function of high endothelial venules [11, 26–30]. The number of SIGLEC1^+^ (CD169) sinus macrophages were also reduced in MLL-LNs [31] further suggesting decreased antigen-presentation in pre-metastatic LNs. Such changes could possibly precondition the LNs for subsequent metastatic growth (microscopically evident 4 days later in this experimental model).

In this study, we now demonstrate that the gene expression pattern in MLL-LNs was separate from the pattern seen in AT1- and vehicle-LNs. A response presumably promoting subsequent metastatic colonization could be detected in regional LNs already 3 days after tumor cell implantation. At this time point a reduction of CD3 (expressed by T lymphocytes) expression was seen both at the protein and mRNA level in MLL-LNs. This was accompanied by increased expression of the immunosuppressive cytokines *Il4* (type 2 driven inflammation) and *Il10* (anti-inflammatory cytokine) [7, 15, 16, 32, 33]. At day 7, we observed a higher expression of *Il2ra* (*Cd25*), *Cd4* and *Foxp3* in MLL-LNs compared to AT1-LNs, suggesting a higher fraction of immunosuppressive regulatory T-cells [34], and also higher *Lyve1* expression suggesting lymphangiogenesis [7, 15, 16]. Expression of *Il6* –a factor promoting a tumor stimulating inflammation and metastasis in prostate cancer [35]–was also increased early (day 3 and 7) in MLL-LNs, as well as *Itgam* (day 3 and 7)–expressed on a diversity of myeloid cells (for example myeloid derived suppressor cells, known to be recruited to pre-metastatic niches) [36].

Notably some factors presumably promoting a T helper type 1-dominated inflammation, like *Tbx21* and *Ifng* [16, 33] were also increased early in MLL-LNs. This suggests that some changes in the LNs could also be part of a defense mechanism against the tumor. However, IFN-gamma could be immunosuppressive when acting together with IL10 [16]. Interestingly, many of the changes seen in pre-metastatic MLL-LNs have also been reported in pre-metastatic LNs draining highly metastatic melanomas [15–17, 37, 38].

At day 10, the macrophage markers *Marco* and *Cd209b* had a higher expression in AT1- vs. MLL-LNs. This could indicate higher amounts of macrophages (important for antigen-presentation within the LNs) and consequently a more efficient anti-tumor T-cell response in the AT1-model at this time-point. Conversely, the expression of *Ido1*, possibly derived from myeloid derived suppressor cells or immature dendritic cells, was higher in MLL- compared to AT1-LNs indicating a suppressed T-cell response [39, 40].

Several differences were also found at day 14, which was the time point when MLL-tumors were larger than AT1 and microscopic metastases were found in the MLL-LNs. These changes could therefore also be related to the presence of micrometastases and/or related to tumor size.

Collectively, our findings suggest that an immunosuppressive environment in regional pre-metastatic LNs could be established through a sequence of changes occurring at different time points. This implies that a single marker can probably not be used to define LNs preparing for subsequent metastatic colonization. Instead, a set of different markers (when better defined) could possibly be used to determine that a LN is preconditioned for subsequent metastatic growth, and at what temporal stage of this process [2].

Regional LNs apparently recognized the presence of small tumors with high metastatic capacity already 3 days after tumor cell injection. At this time point tumors had a diameter of less than 0.6 mm and a volume less than 1% of the ventral prostate lobe volume, corresponding to a <0.5g tumor in a 50g human prostate–a size that is often considered curable [41]. The few MLL tumor cells present at this time point were surrounded by inflammatory cells particularly PMNs and macrophages. Interestingly, tumor-derived factors may convert both PMNs and macrophages into subtypes (N2-PMNs and M2-macrophages) that mediate immunosuppression and metastasis [33, 42, 43]. In our previous studies, we demonstrated active angiogenesis and accumulation of tumor promoting M2- (CD163^+^) macrophages in and around MLL-tumors [9, 19]. We now suggest, in line with data from experimental models of prostate [44] and pancreatic cancer [45] that this is, particularly in aggressive tumors, accompanied by accumulation of metastasis-promoting PMNs [46]. Hypothetically, the signals causing early responses in regional LNs could be derived not only from the few tumor cells present in the primary tumor, but also from accumulating M2-macrophages and N2-PMNs. If so, the tumor-induced inflammation could be necessary to amplify tumor-derived signals and needed to influence remote organs like LNs.

The signals mediating the LN response are unknown (see [11] for discussion). They could be individual secreted factors or many packed together in exosomes. Multiple studies have suggested that tumor-derived exosomes are central in creating pre-metastatic niches in regional lymph nodes [3, 4, 7, 47] in a variety of tumor types suggesting that tumor-derived exosomes could be one signal reaching regional LNs also in prostate cancer. In line with this a single injection of exosomes from highly malignant MLL-tumors into the prostate can pre-condition it for accelerated growth of low malignant tumor cells injected into the prostate three days later [12]. Furthermore, exosomes have also been shown to suppress tumor immunity [48, 49] and could therefore be part of the immune suppression seen in the MLL-model. Further studies are needed to explore the role of exosomes in the draining LNs in this model.

In animals with AT1-tumors, LN changes in mRNA expression pattern and morphology were more similar to those in vehicle-injected animals than to those in animals with MLL-tumors. These alterations are probably signs of an activated anti-tumor immune response restricting and delaying metastatic growth in this otherwise poorly differentiated and rapidly growing tumor type. In patients with other tumors than prostate cancer, increased T-cell number, as well as macrophages, in tumor-free regional LNs is associated with a good prognosis [7, 29, 50]. Further studies of the macrophage- and T-cell populations in regional LNs in prostate cancer patients are therefore warranted.

The question whether particularly aggressive human prostate tumors can create pre-metastatic changes in regional LNs, and if so at what stage of tumor development, is largely unknown. However, some studies of pre-metastatic LNs suggest that the levels of VEGFR1[51, 52], IL30 [53], and CD169 [31] are related to disease progression after prostatectomy. Tumor-free LNs from prostate cancer patients contained more CD68^+^ and pSTAT-3^+^ macrophages than LNs from individuals without prostate cancer [54]. LNs with prostate cancer metastases showed signs of immunosuppression and were often smaller than normal, and these responses occurred prior to arrival of metastatic cells [55].

Collectively, these studies suggest that pre-metastatic, presumably immunosuppressive, changes could be present in patients. Our experimental findings suggest that such changes could potentially be detectable already when tumors are small. However, the time-window when individual pre-metastatic changes (already described and others yet to be discovered) can be detected in patients and how different pre-metastatic markers should be combined for best prediction of subsequent metastatic disease remains to be explored.

## Supporting information

S1 Fig Draining LNs. Black ink was injected into the right ventral prostate and draining lymph nodes were identified.
(TIF)

S1 Table Primers. (DOCX)

S2 Table P-values (MWU and t-test) from comparisons of mRNA expression in vehicle-, AT1-, and MLL-LNs vs. treatment naive-LNs. (DOCX)
