# Supplementary figures and images for: Highly aggressive rat prostate tumors rapidly precondition regional lymph nodes for subsequent metastatic growth
Source: PLoS One. 2017 Oct 26;12(10):e0187086. doi: 10.1371/journal.pone.0187086 (PMC5658154; doi:10.1371/journal.pone.0187086)

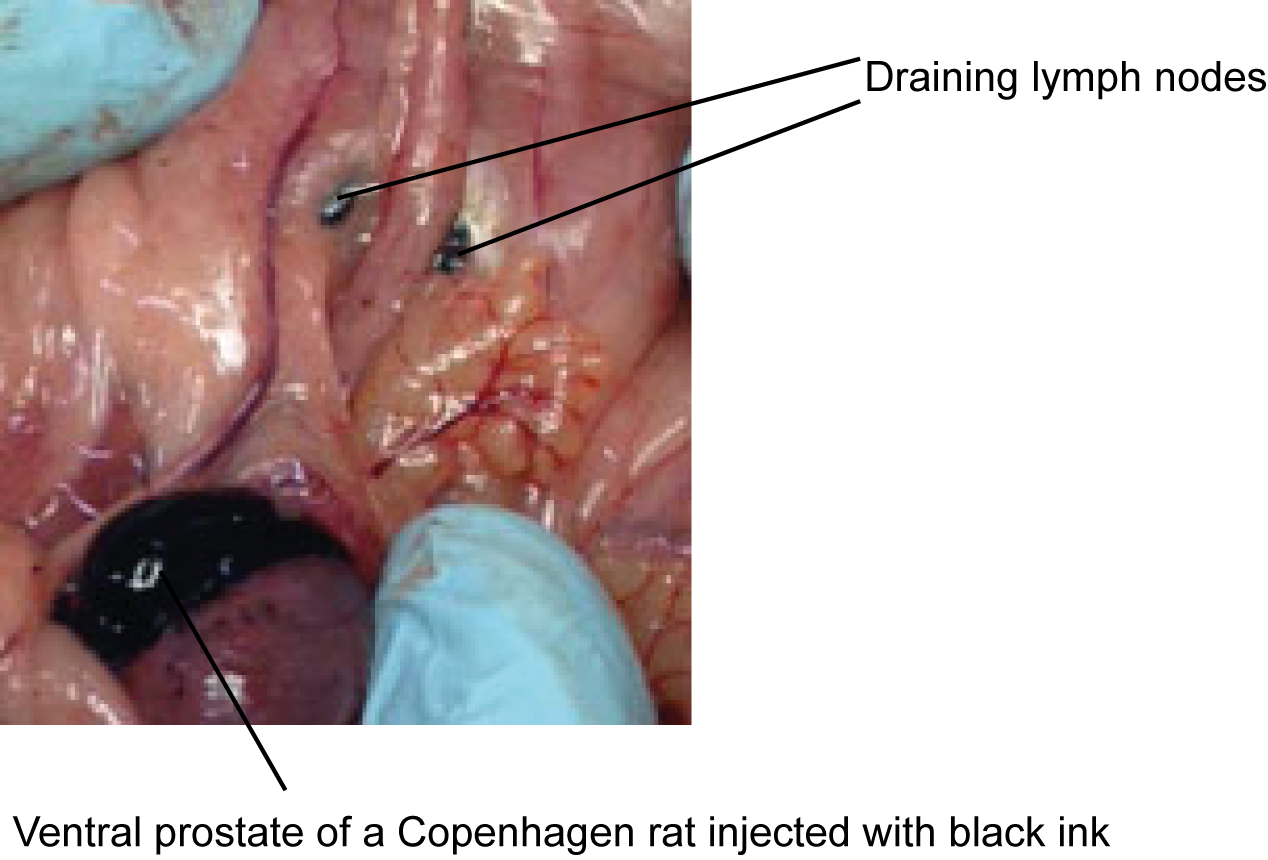

Supplement: S1 Fig — Black ink was injected into the right ventral prostate and draining lymph nodes were identified. (TIF) [file pone.0187086.s001.tif]
