# Supplementary material for: Highly aggressive rat prostate tumors rapidly precondition regional lymph nodes for subsequent metastatic growth
Source: PLoS One. 2017 Oct 26;12(10):e0187086. doi: 10.1371/journal.pone.0187086 (PMC5658154; doi:10.1371/journal.pone.0187086)
Supplement: S1 Table — (DOCX) [file pone.0187086.s002.docx]

| **Supporting Table 1. Primers** | | | | | | |
| --- | --- | --- | --- | --- | --- | --- |
| **Primer Pair ID** | **Gene Symbol** | **RefSeq ID** | **Cat#** | **Oligo#** | **Batch#** | **Sequence: (5' to 3')** |
| R_Cd209b_1 | Cd209b | NM_001170397 | KSPQ12012G | 8019527388-20/0 | HA07854693 | ATGGTTCTACTCTGTCATCC |
|  |  |  | KSPQ12012G | 8019527388-20/1 | HA07854694 | TCGCAGACTTCTTGAAAATC |
| R_Cd3e_1 | Cd3e | NM_001108140 | KSPQ12012G | 8017612374-90/0 | HA06731889 | CATCCTAGAACCTTCCTCTC |
|  |  |  | KSPQ12012G | 8017612374-90/1 | HA06731890 | GATATGGAGATGACATTGGTG |
| R_Cd4_1 | Cd4 | NM_012705 | KSPQ12012G | 8017612374-30/0 | HA06731877 | CCAAGATCCAGGTTTTATCC |
|  |  |  | KSPQ12012G | 8017612374-30/1 | HA06731878 | ACCTGACACAGAATAGGATG |
| R_Cd69_1 | Cd69 | NM_134327 | KSPQ12012G | 8017612374-60/0 | HA06731883 | CACTTCCTTAATTATCGCTCTC |
|  |  |  | KSPQ12012G | 8017612374-60/1 | HA06731884 | TTTCCCGTTGTATGAAAACC |
| R_Cd8a_1 | Cd8a | NM_031538 | KSPQ12012G | 8017612374-20/0 | HA06731875 | CTACTATTTCTGCTCAATCACC |
|  |  |  | KSPQ12012G | 8017612374-20/1 | HA06731876 | AGTTCACTTTCTGAAACACC |
| R_Ctla4_1 | Ctla4 | NM_031674 | KSPQ12012G | 8017612374-160/0 | HA06731903 | CAGATTTATGTCATCGATCCAG |
|  |  |  | KSPQ12012G | 8017612374-160/1 | HA06731904 | AACTGTAGAAAAACAACCCC |
| R_Emr1_1 | Emr1 | NM_001007557 | KSPQ12012G | 8017612374-190/0 | HA06731909 | CTTCACATGCAAAAGGATTC |
|  |  |  | KSPQ12012G | 8017612374-190/1 | HA06731910 | GTTTTCACAGGTGTTATCCAG |
| R_Foxp3_1 | Foxp3 | NM_001108250 | KSPQ12012G | 8017612374-100/0 | HA06731891 | GAAGATGGCATTGACAAAAG |
|  |  |  | KSPQ12012G | 8017612374-100/1 | HA06731892 | CATGTTGTGGAAGAACTCTG |
| R_Gata3_1 | Gata3 | NM_133293 | KSPQ12012G | 8017612374-70/0 | HA06731885 | TAAAATGAATGGGCAGAACC |
|  |  |  | KSPQ12012G | 8017612374-70/1 | HA06731886 | GGGGTCTGTTAATATTGTGG |
| R_Ido1_1 | Ido1 | NM_023973 | KSPQ12012G | 8019527388-10/0 | HA07854691 | ATATTTGTCTGGTTGGAAGG |
|  |  |  | KSPQ12012G | 8019527388-10/1 | HA07854692 | CTTTATTCCCAGAAGGACATC |
| R_Ifng_1 | Ifng | NM_138880 | KSPQ12012G | 8017612374-130/0 | HA06731897 | GAAAGCCTAGAAAGTCTGAAG |
|  |  |  | KSPQ12012G | 8017612374-130/1 | HA06731898 | AGTATTTTCGTGTTACCGTC |
| R_Il10_1 | Il10 | NM_012854 | KSPQ12012G | 8017612374-120/0 | HA06731895 | TCTCCCCTGTGAGAATAAAAG |
|  |  |  | KSPQ12012G | 8017612374-120/1 | HA06731896 | TAGACACCTTTGTCTTGGAG |
| R_Il2ra_1 | Il2ra | NM_013163 | KSPQ12012G | 8017612374-50/0 | HA06731881 | AACATAGATGGAGGAAGAGC |
|  |  |  | KSPQ12012G | 8017612374-50/1 | HA06731882 | GACTTCATAACTTTCCAGGAC |
| R_Il4_1 | Il4 | NM_201270 | KSPQ12012G | 8017612374-170/0 | HA06731905 | GAACCAGGTCACAGAAAAAG |
|  |  |  | KSPQ12012G | 8017612374-170/1 | HA06731906 | GGGAAGTAAAATTTGCGAAG |
| R_Il6_1 | Il6 | NM_012589 | KSPQ12012G | 8017612374-10/0 | HA06731873 | CAGAGTCATTCAGAGCAATAC |
|  |  |  | KSPQ12012G | 8017612374-10/1 | HA06731874 | CTTTCAAGATGAGTTGGATGG |
| R_Itgam_1 | Itgam | NM_012711 | KSPQ12012G | 8017612374-40/0 | HA06731879 | GTAGTGAGAGAACTGTTTCAG |
|  |  |  | KSPQ12012G | 8017612374-40/1 | HA06731880 | TCCTCATAATTTAGGGGGTC |
| R_Lyve1_1 | Lyve1 | NM_001106286 | KSPQ12012G | 8019527388-30/0 | HA07854695 | CTACGTGAAAAGGTATGTGAAG |
|  |  |  | KSPQ12012G | 8019527388-30/1 | HA07854696 | CATCATCAGCTTTCTCTTCC |
| R_Marco_1 | Marco | NM_001109011 | KSPQ12012G | 8017612374-200/0 | HA06731911 | GTGATGGAGACCTTTGAAATC |
|  |  |  | KSPQ12012G | 8017612374-200/1 | HA06731912 | TTGAGAACTTGAATCAGCAG |
| R_Pi4Kb_1 | Pi4kb | NM_031083 | KSPQ12012G | 8018655924-50/0 | HA07358495 | CTATTACAAGTCAAGGACAGG |
|  |  |  | KSPQ12012G | 8018655924-50/1 | HA07358496 | GTTGAACATATCACCGTTCAG |
| R_Rab14_1 | Rab14 | NM_053589 | KSPQ12012G | 8018655924-10/0 | HA07358487 | CAGAACATTCAGGATGGAAG |
|  |  |  | KSPQ12012G | 8018655924-10/1 | HA07358488 | GTGAAAGGTCAAAATGAGGG |
| R_RGD1311578_1 | RGD1311578 | NM_001008318 | KSPQ12012G | 8018655924-60/0 | HA07358497 | ATACAACGCGATGAAAGAAG |
|  |  |  | KSPQ12012G | 8018655924-60/1 | HA07358498 | GTTCATAAAACCCTCTCAAGG |
| R_Tbx21_1 | Tbx21 | NM_001107043 | KSPQ12012G | 8017612374-80/0 | HA06731887 | AAAGCTTACCAACAACAAGG |
|  |  |  | KSPQ12012G | 8017612374-80/1 | HA06731888 | CTTGGAAGGTAAAGATGTGAG |
| R_Tgfb1_1 | Tgfb1 | NM_021578 | KSPQ12012G | 8017612374-150/0 | HA06731901 | GGAAATCAATGGGATCAGTC |
|  |  |  | KSPQ12012G | 8017612374-150/1 | HA06731902 | CTGAAGCAGTAGTTGGTATC |
