# Supplementary material for: Highly aggressive rat prostate tumors rapidly precondition regional lymph nodes for subsequent metastatic growth
Source: PLoS One. 2017 Oct 26;12(10):e0187086. doi: 10.1371/journal.pone.0187086 (PMC5658154; doi:10.1371/journal.pone.0187086)
Supplement: S2 Table — (DOCX) [file pone.0187086.s003.docx]

| **Supporting table 2. P-values (MWU and t-test) from comparisons of mRNA expression in vehicle-, AT1-, and MLL-LNs vs. treatment naive-LNs*** | | | | | | | | | | | | | |
| --- | --- | --- | --- | --- | --- | --- | --- | --- | --- | --- | --- | --- | --- |
|  |  | **Day 3** | | | **Day 7** | | | **Day 10** | | | **Day 14** | | |
|  |  | **Vehicle** | **AT1** | **MLL** | **Vehicle** | **AT1** | **MLL** | **Vehicle** | **AT1** | **MLL** | **Vehicle** | **AT1** | **MLL** |
| ***Cd209b*** | MWU | 0.385 | 0.452 | 0.224 | 0.024 | 0.007 | 0.073 | 0.751 | 0.073 | 0.138 | 0.183 | 0.685 | 0.250 |
|  | T-test | 0.152 | 0.446 | 0.145 | 0.013 | 0.004 | 0.026 | 0.323 | 0.166 | 0.080 | 0.089 | 0.616 | 0.134 |
| ***Cd3*** | MWU | 0.066 | 0.010 | 0.031 | 0.041 | 0.083 | 0.002 | 0.011 | 0.227 | 0.001 | 0.001 | 0.001 | 0.183 |
|  | T-test | 0.073 | 0.003 | 0.017 | 0.019 | 0.071 | 0.000 | 0.004 | 0.191 | 0.000 | 0.000 | 0.000 | 0.234 |
| ***Cd4*** | MWU | 0.024 | 0.156 | 0.031 | 0.010 | 0.001 | 0.014 | 0.112 | 0.128 | 0.018 | 0.002 | 0.002 | 0.385 |
|  | T-test | 0.035 | 0.085 | 0.024 | 0.004 | 0.000 | 0.005 | 0.208 | 0.259 | 0.009 | 0.000 | 0.000 | 0.484 |
| ***Cd69*** | MWU | 0.043 | 0.183 | 0.056 | 0.862 | 0.862 | 0.325 | 0.001 | 0.452 | 0.290 | 0.385 | 0.452 | 0.011 |
|  | T-test | 0.045 | 0.120 | 0.018 | 0.916 | 0.975 | 0.265 | 0.000 | 0.274 | 0.248 | 0.225 | 0.346 | 0.006 |
| ***Cd8*** | MWU | 0.958 | 0.637 | 0.128 | 0.024 | 0.002 | 0.014 | 0.024 | 0.083 | 0.006 | 0.001 | 0.004 | 0.032 |
|  | T-test | 0.463 | 0.200 | 0.209 | 0.014 | 0.000 | 0.005 | 0.014 | 0.359 | 0.002 | 0.000 | 0.000 | 0.006 |
| ***Ctla4*** | MWU | 0.001 | 0.001 | 0.002 | 0.002 | 0.001 | 0.001 | 0.008 | 0.018 | 0.049 | 0.001 | 0.001 | 0.005 |
|  | T-test | 0.000 | 0.000 | 0.000 | 0.001 | 0.000 | 0.000 | 0.008 | 0.016 | 0.036 | 0.000 | 0.000 | 0.001 |
| ***Emr1*** | MWU | 0.003 | 0.001 | 0.862 | 0.603 | 0.073 | 0.272 | 0.290 | 0.954 | 0.169 | 0.007 | 0.272 | 0.030 |
|  | T-test | 0.000 | 0.000 | 0.877 | 0.863 | 0.049 | 0.090 | 0.187 | 0.972 | 0.137 | 0.002 | 0.292 | 0.022 |
| ***Foxp3*** | MWU | 0.056 | 0.056 | 0.685 | 0.001 | 0.001 | 0.148 | 0.751 | 0.183 | 0.525 | 0.001 | 0.001 | 0.160 |
|  | T-test | 0.090 | 0.038 | 0.754 | 0.000 | 0.000 | 0.139 | 0.887 | 0.229 | 0.618 | 0.000 | 0.000 | 0.068 |
| ***Gata3*** | MWU | 0.118 | 0.524 | 0.325 | 0.013 | 0.002 | 0.118 | 0.290 | 0.272 | 0.397 | 0.001 | 0.002 | 0.041 |
|  | T-test | 0.090 | 0.632 | 0.445 | 0.015 | 0.000 | 0.061 | 0.226 | 0.183 | 0.303 | 0.000 | 0.000 | 0.025 |
| ***Ido*** | MWU | 0.001 | 0.001 | 0.013 | 0.001 | 0.001 | 0.001 | 0.020 | 0.024 | 0.002 | 0.001 | 0.001 | 0.002 |
|  | T-test | 0.000 | 0.000 | 0.005 | 0.000 | 0.000 | 0.000 | 0.028 | 0.047 | 0.001 | 0.000 | 0.000 | 0.000 |
| ***Ifng*** | MWU | 0.002 | 0.003 | 0.005 | 0.118 | 0.093 | 0.001 | 0.004 | 0.018 | 0.090 | 0.118 | 0.093 | 0.055 |
|  | T-test | 0.000 | 0.000 | 0.004 | 0.120 | 0.064 | 0.000 | 0.001 | 0.011 | 0.078 | 0.143 | 0.108 | 0.109 |
| ***Il10*** | MWU | 0.138 | 0.949 | 0.004 | 0.846 | 0.401 | 0.004 | 0.138 | 0.006 | 0.045 | 0.747 | 0.747 | 0.008 |
|  | T-test | 0.085 | 0.953 | 0.003 | 0.850 | 0.242 | 0.001 | 0.070 | 0.002 | 0.050 | 0.449 | 0.656 | 0.001 |
| ***Il2ra*** | MWU | 0.001 | 0.003 | 0.118 | 0.002 | 0.003 | 0.272 | 0.112 | 0.073 | 0.751 | 0.452 | 0.524 | 0.011 |
|  | T-test | 0.000 | 0.001 | 0.280 | 0.000 | 0.000 | 0.130 | 0.067 | 0.081 | 0.342 | 0.578 | 0.462 | 0.007 |
| ***Il4*** | MWU | na | na | na | na | na | na | na | na | na | na | na | na |
|  | T-test | 0.416 | 0.384 | 0.004 | 0.365 | 0.078 | 0.006 | 0.030 | 0.255 | 0.082 | 0.007 | 0.022 | 0.220 |
| ***Il6*** | MWU | 0.003 | 0.009 | 0.001 | 0.452 | 0.073 | 0.001 | 0.009 | 0.772 | 0.290 | 0.056 | 0.056 | 0.041 |
|  | T-test | 0.002 | 0.001 | 0.001 | 0.341 | 0.047 | 0.000 | 0.003 | 0.637 | 0.313 | 0.038 | 0.050 | 0.040 |
| ***Itgam*** | MWU | 0.603 | 0.183 | 0.009 | 0.183 | 0.603 | 0.018 | 0.751 | 0.093 | 0.090 | 0.001 | 0.002 | 0.125 |
|  | T-test | > 0.99 | 0.238 | 0.007 | 0.183 | 0.409 | 0.006 | 0.640 | 0.066 | 0.058 | 0.000 | 0.000 | 0.141 |
| ***Lyve1*** | MWU | 0.002 | 0.002 | 0.002 | 0.009 | 0.002 | 0.685 | 0.597 | 0.148 | 0.916 | 0.009 | 0.093 | 0.701 |
|  | T-test | 0.000 | 0.000 | 0.000 | 0.008 | 0.000 | 0.684 | 0.910 | 0.121 | 0.711 | 0.004 | 0.124 | 0.765 |
| ***Marco*** | MWU | 0.007 | 0.093 | 0.118 | 0.032 | 0.003 | 0.224 | 0.751 | 0.043 | 0.916 | 0.148 | 0.862 | 0.609 |
|  | T-test | 0.003 | 0.035 | 0.127 | 0.022 | 0.001 | 0.129 | 0.614 | 0.025 | 0.816 | 0.090 | 0.598 | 0.565 |
| ***Tbx21*** | MWU | 0.183 | 0.183 | 0.005 | 0.603 | 0.685 | 0.001 | 0.011 | 0.183 | 0.072 | 0.093 | 0.224 | 0.250 |
|  | T-test | 0.098 | 0.174 | 0.000 | 0.489 | 0.237 | 0.000 | 0.002 | 0.140 | 0.042 | 0.068 | 0.063 | 0.330 |
| ***Tgfb1*** | MWU | 0.001 | 0.001 | 0.013 | 0.005 | 0.954 | 0.032 | 0.341 | 0.001 | 0.002 | 0.001 | 0.001 | 0.002 |
|  | T-test | 0.000 | 0.000 | 0.014 | 0.000 | 0.945 | 0.012 | 0.418 | 0.000 | 0.000 | 0.000 | 0.000 | 0.000 |
| * log2 mean relative expression is shown in Fig 6. Mann Whitney U (MWU), not applicable (na). | | | | | | | | | | | | | |
